# Low handgrip strength is associated with impaired gait and dexterity in patients with degenerative cervical myelopathy: A cross-sectional study

**DOI:** 10.1097/MD.0000000000050294

**Published:** 2026-08-21

**Authors:** Hirokazu Inoue, Hideaki Sawamura, Hideaki Watanabe, Hitoshi Okami, Yasuyuki Shiraishi, Atsushi Kimura, Katsushi Takeshita, Mitsuya Morita

**Affiliations:** a Rehabilitation Center, Jichi Medical University Hospital, Shimotsuke, Japan; b Department of Orthopaedics, Jichi Medical University, Shimotsuke, Japan; c Department of Pediatric Orthopaedic Surgery, Jichi Children’s Medical Center, Shimotsuke, Japan; d Department of Orthopaedic Surgery, Shinkaminokawa Hospital, Kaminokawa, Japan.

**Keywords:** cervical myelopathy, dexterity, frailty, handgrip strength, pinch strength

## Abstract

Degenerative cervical myelopathy (DCM) is the most common cause of adult spinal cord dysfunction and leads to progressive neurological deficits in gait and hand dexterity. Handgrip strength is a recognized marker of overall muscle strength and has been linked to various clinical outcomes, including frailty and prognosis. This study aimed to evaluate the relationship between handgrip strength, frailty, and functional status in DCM patients. This cross-sectional study included 94 patients (65 males, 29 females; mean age 66.9 years) undergoing surgery for DCM. Preoperatively, we measured handgrip strength, lower extremity extension power (LEP), pinch strength, gait speed (10-m walk test), hand dexterity (counter test), and neurological function (Japanese Orthopaedic Association [JOA] score). We also assessed frailty using the 5-item modified frailty index (mFI-5) and nutritional status using the Geriatric Nutritional Risk Index (GNRI). Handgrip strength showed strong positive correlations with LEP (*r* = 0.675, *P* < .001), gait speed (*r* = 0.617, *P* < .001), hand dexterity (*r* = 0.659, *P* < .001), and pinch strength (*r* = 0.723, *P* < .001). It was also positively correlated with the total JOA score (*r* = 0.468, *P* = .001) and negatively correlated with age (r = −0.468, *P* < .001) and the mFI-5 score (r = −0.464, *P* < .001). Handgrip strength was significantly lower in frail patients (19.5 ± 9.7 kg) than in prefrail (25.2 ± 7.1 kg) and robust patients (30.0 ± 7.5 kg) (*P* < .001). After adjustment for age and sex, the associations with LEP (partial *r* = 0.430, *P* < .001), hand dexterity (partial *r* = 0.446, *P* < .001), and pinch strength (partial *r* = 0.560, *P* < .001) remained significant after Bonferroni correction, whereas the association with gait speed did not (partial *r* = 0.257, *P* = .030). Handgrip strength was associated with gait performance, dexterity, neurological function, and frailty in unadjusted analyses. After adjustment for age and sex, the strongest adjusted associations were with LEP and hand function. Handgrip strength may complement comprehensive preoperative assessment, although prospective validation is required before it can guide clinical management.

## 1. Introduction

Degenerative cervical myelopathy (DCM) is the most common cause of adult spinal cord dysfunction and is characterized by progressive neurological deficits, including gait disturbance and impaired hand dexterity.^[1–3]^ Given that DCM primarily affects middle-aged and older adults, understanding the impact of aging-related conditions such as frailty on patient outcomes has become a critical area of clinical investigation.^[4,5]^

Frailty represents a state of decreased physiological reserves and increased vulnerability to adverse health outcomes. It is closely linked to sarcopenia, a condition defined by the loss of muscle mass and function, including reduced handgrip strength and walking speed.^[6,7]^ A decline in handgrip strength is now a widely accepted component of frailty assessment and is considered a robust, useful, and simple indicator of overall muscle strength and nutritional status.^[8,9]^ While the link between handgrip strength and frailty is well-established in general geriatric and non-neurological patient cohorts, its relevance in DCM warrants specific investigation. In DCM, spinal cord compression can lead to both upper extremity weakness (directly impacting grip) and lower extremity dysfunction, suggesting that handgrip strength may reflect the systemic and neurological impact of the disease rather than just localized hand function.^[1–3]^

While numerous studies have reported on the epidemiology, treatment outcomes, and functional deficits in DCM patients,^[10,11]^ the role of handgrip strength as a simple, objective screening tool for DCM has received limited attention. Handgrip strength has been shown to be a valuable predictor of fall risk and clinical outcomes in other spinal conditions such as lumbar spinal stenosis, where it correlates with walking function.^[12,13]^ Furthermore, recent studies have begun to explore the use of handgrip strength, both alone and in combination with dexterity tests, as a diagnostic and prognostic tool for DCM.^[14,15]^ Importantly, handgrip strength has been specifically correlated with pre- and postoperative fall risk and functional outcomes in patients with DCM, highlighting its potential clinical utility.^[16]^ However, patients with DCM exhibit a unique neurological profile, with gait and hand function deficits directly stemming from spinal cord compression, making the potential correlation with handgrip strength particularly relevant.^[15–17]^

This study aimed to examine the relationship between handgrip strength, gait speed, hand dexterity, and frailty in a cohort of patients with degenerative cervical myelopathy. We hypothesized that handgrip strength is significantly correlated with key functional measures and can serve as a simple, noninvasive screening tool for functional status in DCM patients, providing valuable insight into their overall physical condition and potential for frailty. This study aimed to clarify the association between handgrip strength measurement and functional status in the preoperative assessment of patients with DCM.

## 2. Materials and methods

### 2.1. Study sample

We retrospectively enrolled patients who had undergone cervical spine surgery at a single hospital between June 2015 and March 2018. This study was approved by the Ethics Review Board of Shinkaminokawa Hospital. Informed consent was obtained from all participants. This study was conducted in accordance with the principles of the World Medical Association Declaration of Helsinki. We calculated the necessary sample size for the study to achieve an alpha value of 0.05 and a power of 0.80 using G*Power 3 statistical software (v 3.1.9.7, Heinrich-Heine-University, Düsseldorf, Germany),^[18,19]^ resulting in an estimated sample size of 82. All patients exhibited symptoms of myelopathy, including hand dexterity, poor hand coordination (e.g., difficulty in writing and using chopsticks), and walking difficulty. The exclusion criteria were comorbidities that impaired physical function (e.g., stroke, cerebral palsy, and severe rheumatoid arthritis), a history of spinal surgery, and difficulty completing the questionnaire because of cognitive impairment.

### 2.2. Measurements

All patients underwent all measurements before surgery. The handgrip strength of both upper extremities was measured using a handheld dynamometer. The tests were performed twice (on both the right and left hands) with a short break between each test. The best performance was chosen for the analysis.^[13]^ Leg extension power (LEP) was measured with participants in a seated position with the knee and hip flexed at 90 degrees, using an isokinetic leg power system (µTas F-1; Anima, Tokyo, Japan). Standardized verbal encouragement was provided during each trial, instructing participants to “push as hard and as fast as you can.” While laboratory protocols may involve more repetitions, 2 trials represent a standard clinical practice to minimize patient fatigue. The best performance was used for the analysis, consistent with established protocols. Walking speed was measured twice in the 10-meter walking test, and the best result was used. For patients who were unable to complete the 10-m walk test, walking speed was recorded as 0 m/s. The counter test (number of button pushes in 10 seconds) was performed using a hand tally counter (Kokuyo, Osaka, Japan).^[20]^ The counts were recorded twice bilaterally, and the best performance was used for the analysis. Bilateral pinch strength was assessed using a pinch gauge (SPR-641; Sakai Iryo Co., Tokyo, Japan) at the tip pinch positions. Two trials were performed in both the right and left hands, with a short break between trials, and the best results were used in the analysis.

The Japanese Orthopaedic Association (JOA) score for rating cervical myelopathy assesses 6 categories of cervical myelopathy: motor dysfunction of the upper and lower extremities, sensory dysfunction of the upper and lower extremities and trunk, and bladder function. These subscales are scored on a scale of 0 − 4, 4, 2, 2, 2, and 3, respectively, with a minimum score of 0 and a maximum score of 17. Patients were categorized by frailty status as robust, prefrail, or frail based on the 5-item modified frailty index (mFI-5) (robust: mFI = 0, prefrail: mFI = 1, frail: mFI ≥ 2).^[9]^ The mFI-5 consists of 5 National Surgical Quality Improvement Program (NSQIP) variables: history of severe chronic obstructive pulmonary disease, congestive heart failure within 30 days before surgery, functional health status before admission, hypertension requiring medication, and diabetes mellitus with oral agents or insulin.

The Geriatric Nutritional Risk Index (GNRI) was calculated using the following formula: GNRI = 14.89 × preoperative serum albumin (g/dL) + 41.7 × (current body weight [kg]/ideal body weight [kg]).^[21]^ The ideal body weight was calculated as (height [m])^2^ × 22.

### 2.3. Comorbidities

Comorbidities were determined based on the laboratory results of blood samples as follows: anemia (hemoglobin ≤ 13 g/dL in males and ≤ 12 g/dL in females), diabetes (blood glucose ≥ 126 mg/dL), hypercholesterolemia (total cholesterol ≥ 190 mg/dL), altered high-density lipoprotein (HDL) cholesterol (<40 mg/dL in males and < 50 mg/dL in females), and hypertriglyceridemia (triglycerides ≥ 150 mg/dL). The use of medication for the treatment of each disease was also considered. Dyslipidemia was defined as triglycerides ≥ 150 mg/dL, low-density lipoprotein (LDL) cholesterol ≥ 160 mg/dL, and HDL cholesterol < 40 mg/dL (males) or < 50 mg/dL (females). Hypertension was defined as the mean of the second and third blood pressure measurements (systolic blood pressure ≥ 140 mm Hg and/or diastolic blood pressure ≥ 90 mm Hg) or the need for hypotensive medication. Body mass index (BMI) was estimated based on weight and height. The low-weight BMI-specific cutoff point was < 22.

### 2.4. Statistical analysis

Continuous variables are expressed as the mean ± standard deviation or median with interquartile range, as appropriate. Unadjusted associations between handgrip strength and the other variables were assessed using Pearson’s correlation coefficient. To account for multiple comparisons, Bonferroni correction was applied separately within each family of analyses and each patient cohort. For the 18 Pearson correlation analyses, statistical significance was defined as *P* < .0028 (0.05/18). Partial correlation analyses controlling for age and sex were subsequently performed for the remaining 16 variables; age and sex were excluded from these analyses because they served as covariates. Age and sex were selected a priori because of their established associations with muscle strength and physical function. For the 16 partial correlation analyses, statistical significance was defined as *P* < .0031 (0.05/16). Unadjusted P values are reported in Tables 1 and 2, and symbols indicate associations that remained statistically significant after Bonferroni correction. The Mann-Whitney U-test was used to compare handgrip strength between patients with and without anemia, diabetes, hypertension, hypercholesterolemia, altered HDL cholesterol, hypertriglyceridemia, dyslipidemia, low weight BMI, and marital status. To account for these 9 comparisons, Bonferroni correction was applied, and statistical significance was defined as *P* < .0056 (0.05/9). Differences in handgrip strength among the robust, prefrail, and frail groups were assessed using the Kruskal–Wallis test followed by Bonferroni-adjusted pairwise comparisons. All statistical analyses were performed using statistical software (SPSS for Windows v17.0; SPSS, Chicago, IL, USA).

**Table 1:** Clinical characteristics of ambulatory patients with degenerative cervical myelopathy.

	Mean (SD)	Pearson correlation		Partial correlation	
		r value	*P* value	r value	*P* value
Age (years)	65.7 ± 12.3	−0.460	<.001*	–	–
Sex (Male: Female)	56:22	−0.621	<.001*	–	–
Height (cm)	162.9 ± 8.5	0.678	<.001*	0.283	.013
Weight (kg)	64.3 ± 13.4	0.478	<.001*	0.247	.031
BMI (kg/m^2^)	24.0 ± 3.8	0.197	.093	0.154	.185
Handgrip strength (kg)	25.9 ± 7.7	–	–	–	–
Leg extension power (kg)	30.7 ± 14.4	0.651	<.001*	0.395	<.001**
Walking speed (m/s)	1.01 ± 0.34	0.478	<.001*	0.257	.030
Counter test	38.3 ± 10.9	0.613	<.001*	0.433	<.001**
Pinch strength (kg)	5.0 ± 2.1	0.695	<.001*	0.568	<.001**
JOA score Total	11.5 ± 3.5	0.315	.037	0.173	.273
Upper extremity motor function	2.4 ± 0.9	0.455	.001*	0.269	.062
Lower extremity motor function	1.9 ± 0.9	0.595	<.001*	0.395	.004
Upper extremity sensory function	0.4 ± 0.6	0.020	.891	−0.088	.546
Lower extremity sensory function	0.9 ± 0.9	−0.188	.217	−0.069	.660
Trunk sensory function	1.8 ± 0.6	−0.267	.087	−0.376	.017
Bladder function	2.4 ± 0.8	−0.096	.561	0.144	.395
mFI-5	1.17 ± 0.97	−0.369	.001*	−0.133	.252
GNRI	108.0 ± 11.3	0.212	.062	0.154	.185

BMI = body mass index, GNRI = geriatric nutritional risk index, JOA = Japanese Orthopaedic Association, mFI-5 = 5-item modified frailty index, SD = standard deviation.

Values are presented as the mean (standard deviation). Unadjusted P values are shown. Pearson r represents the unadjusted correlation coefficient, and partial r represents the correlation coefficient adjusted for age and sex.

* *P* <.0028 after Bonferroni correction for 18 Pearson correlation analyses.

** *P* <.0031 after Bonferroni correction for 16 partial correlation analyses.

**Table 2:** Clinical characteristics of all patients with degenerative cervical myelopathy.

	Mean (SD)	Pearson correlation		Partial correlation	
		r value	*P* value	r value	*P* value
Age (years)	66.9 ± 12.5	−0.468	<.001*	–	–
Sex (Male: Female)	65:29	−0.617	<.001*	–	–
Height (cm)	162.2 ± 8.9	0.601	<.001*	0.116	.272
Weight (kg)	63.7 ± 13.1	0.459	<.001*	0.142	.177
BMI (kg/m^2^)	24.0 ± 3.7	0.198	.056	0.090	.391
Handgrip strength (kg)	23.9 ± 9.3	–	–	–	–
Leg extension power (kg)	29.5 ± 14.7	0.675	<.001*	0.430	<.001**
Walking speed (m/s)	0.83 ± 0.50	0.617	<.001*	0.257	.030
Counter test	37.4 ± 11.2	0.659	<.001*	0.446	<.001**
Pinch strength (kg)	4.8 ± 2.1	0.723	<.001*	0.560	<.001**
JOA score Total	11.0 ± 3.7	0.468	.001*	0.288	.050
Upper extremity motor function	2.2 ± 1.0	0.616	<.001*	0.416	.001**
Lower extremity motor function	1.5 ± 1.1	0.658	<.001*	0.541	<.001**
Upper extremity sensory function	0.3 ± 0.6	0.181	.163	0.007	.956
Lower extremity sensory function	0.8 ± 0.9	0.002	.991	0.096	.505
Trunk sensory function	1.8 ± 0.6	−0.203	.167	−0.248	.097
Bladder function	2.3 ± 0.8	0.085	.572	0.230	.132
mFI-5	1.35 ± 1.01	−0.464	<.001*	−0.273	.008
GNRI	106.7 ± 12.6	0.207	.046	0.131	.214

BMI = body mass index, GNRI = geriatric nutritional risk index, JOA = Japanese Orthopaedic Association, mFI-5 = 5-item modified frailty index, SD = standard deviation.

Values are presented as the mean (standard deviation). Unadjusted P values are shown. Pearson r represents the unadjusted correlation coefficient, and partial r represents the correlation coefficient adjusted for age and sex.

* *P* <.0028 after Bonferroni correction for 18 Pearson correlation analyses.

** *P* <.0031 after Bonferroni correction for 16 partial correlation analyses.

## 3. Results

A total of 102 patients underwent cervical spinal surgery. Eight patients were excluded from the study. Ninety-four patients were enrolled in the study, of which 78 were able to walk and 16 were unable to walk. Cervical spondylotic myelopathy and ossification of the posterior longitudinal ligament were reported in 79 and 15 patients, respectively.

For DCM patients, excluding those who were unable to walk, all parameters were compared with handgrip strength (Table 1). The mean patient age was 65.7 years, and the study population included 56 males and 22 females. There was a significant difference between the sexes (r = −0.621, *P*< .001). Males had a significantly higher handgrip strength than females (*P*< .001; 28.9 ± 6.5 kg vs. 18.4 ± 4.6 kg). Handgrip strength was significantly correlated with age (r = −0.460, *P*< .001), height (r = 0.678, *P*< .001), weight (r = 0.478, *P*< .001), LEP (r = 0.651, *P*< .001), walking speed (r = 0.478, *P*< .001), counter test (r = 0.613, *P*< .001), pinch strength (r = 0.695, *P*< .001), upper extremity motor function (r = 0.455, *P* = .001), lower extremity motor function (r = 0.595, *P*< .001), and mFI-5 (r = −0.369, *P* = .001). The correlations with BMI, the total JOA score, sensory and bladder function, and GNRI did not meet the Bonferroni-corrected significance threshold. After adjustment for age and sex, handgrip strength remained significantly correlated with LEP (partial *r* = 0.395, *P* < .001), counter test performance (partial *r* = 0.433, *P* < .001), and pinch strength (partial *r* = 0.568, *P* < .001) after Bonferroni correction. The adjusted association with walking speed did not meet the Bonferroni-corrected significance threshold (partial *r* = 0.257, *P* = .030).

**Figure 1.**
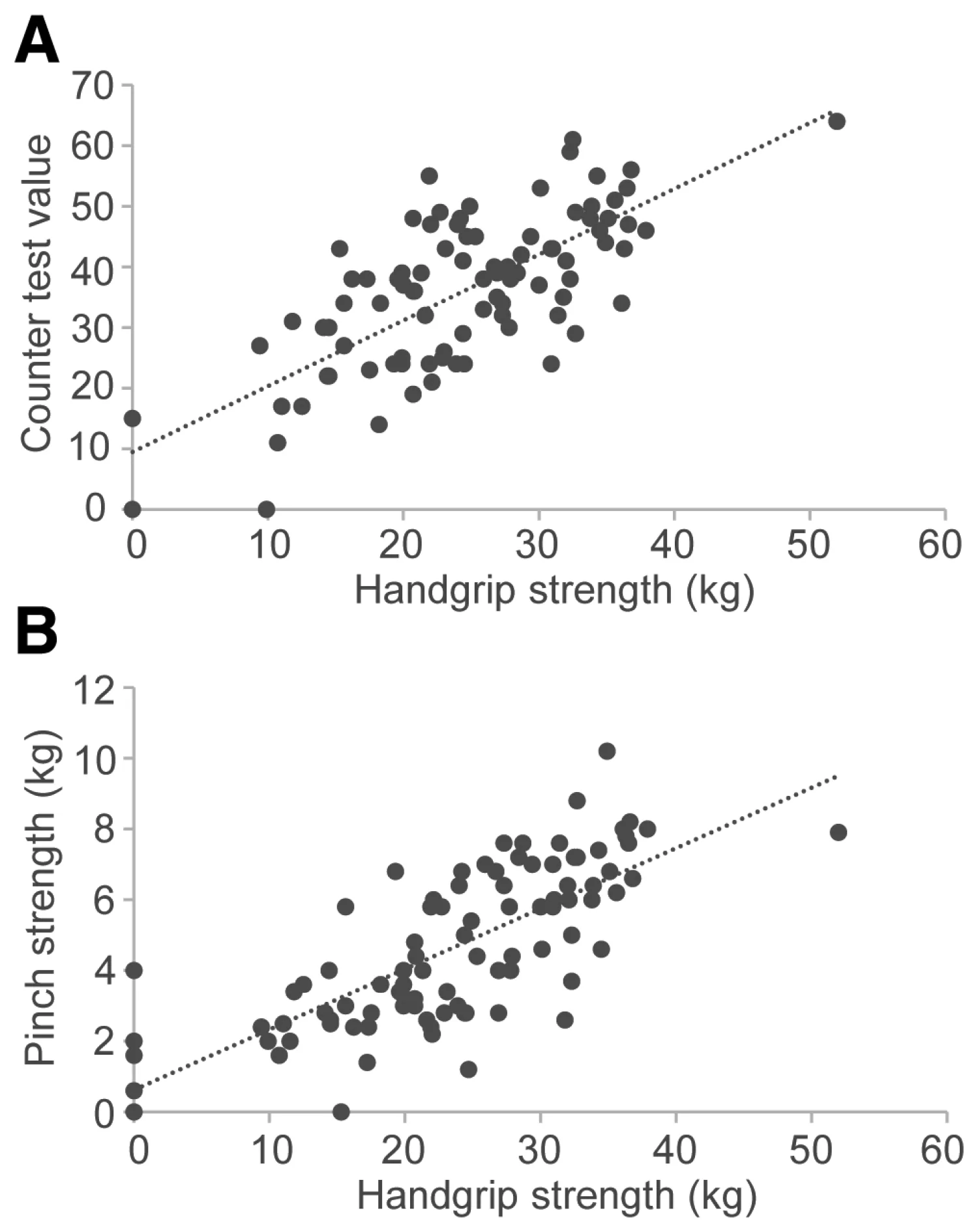
Relationships of handgrip strength with hand dexterity and pinch strength. (A) Counter test performance was positively correlated with handgrip strength (*r* = 0.659, *P* < .001). (B) Pinch strength was positively correlated with handgrip strength (*r* = 0.723, *P* < .001).

Next, for all DCM patients, including those who were unable to walk, all parameters were compared with handgrip strength (Table 2). The mean patient age was 66.9 years, and the study population included 65 males and 29 females. There were significant differences between the sexes (r=−0.617, *P* < .001). Males had a significantly higher handgrip strength than females (*P* < .001; 27.7 ± 7.2 kg vs 15.3 ± 7.7 kg). Handgrip strength was significantly correlated with age (r=−0.468, *P* < .001), height (*r* = 0.601, *P* < .001), weight (*r* = 0.459, *P* < .001), LEP (*r* = 0.675, *P* < .001), walking speed (*r* = 0.617, *P* < .001), counter test (*r* = 0.659, *P* < .001; Fig. 1A), pinch strength (*r* = 0.723, *P* < .001; Fig 1B), total JOA score (*r* = 0.468, *P* = .001), upper extremity motor function (*r* = 0.616, *P* < .001), lower extremity motor function (*r* = 0.658, *P* < .001), and mFI-5 (r=−0.464, *P* < .001). The correlations with BMI, sensory and bladder function, and GNRI did not meet the Bonferroni-corrected significance threshold. After adjustment for age and sex, handgrip strength remained significantly correlated with LEP (partial *r* = 0.430, *P* < .001), counter test performance (partial *r* = 0.446, *P* < .001), pinch strength (partial *r* = 0.560, *P* < .001), upper extremity motor function (partial *r* = 0.416, *P* = .001), and lower extremity motor function (partial *r* = 0.541, *P* < .001) after Bonferroni correction. The adjusted associations with walking speed (partial *r* = 0.257, *P* = .030), total JOA score (partial *r* = 0.288, *P* = .050), and mFI-5 (partial r =−0.273, *P* = .008) did not meet the Bonferroni-corrected significance threshold.

### 3.1. Frailty

**Figure 2.**
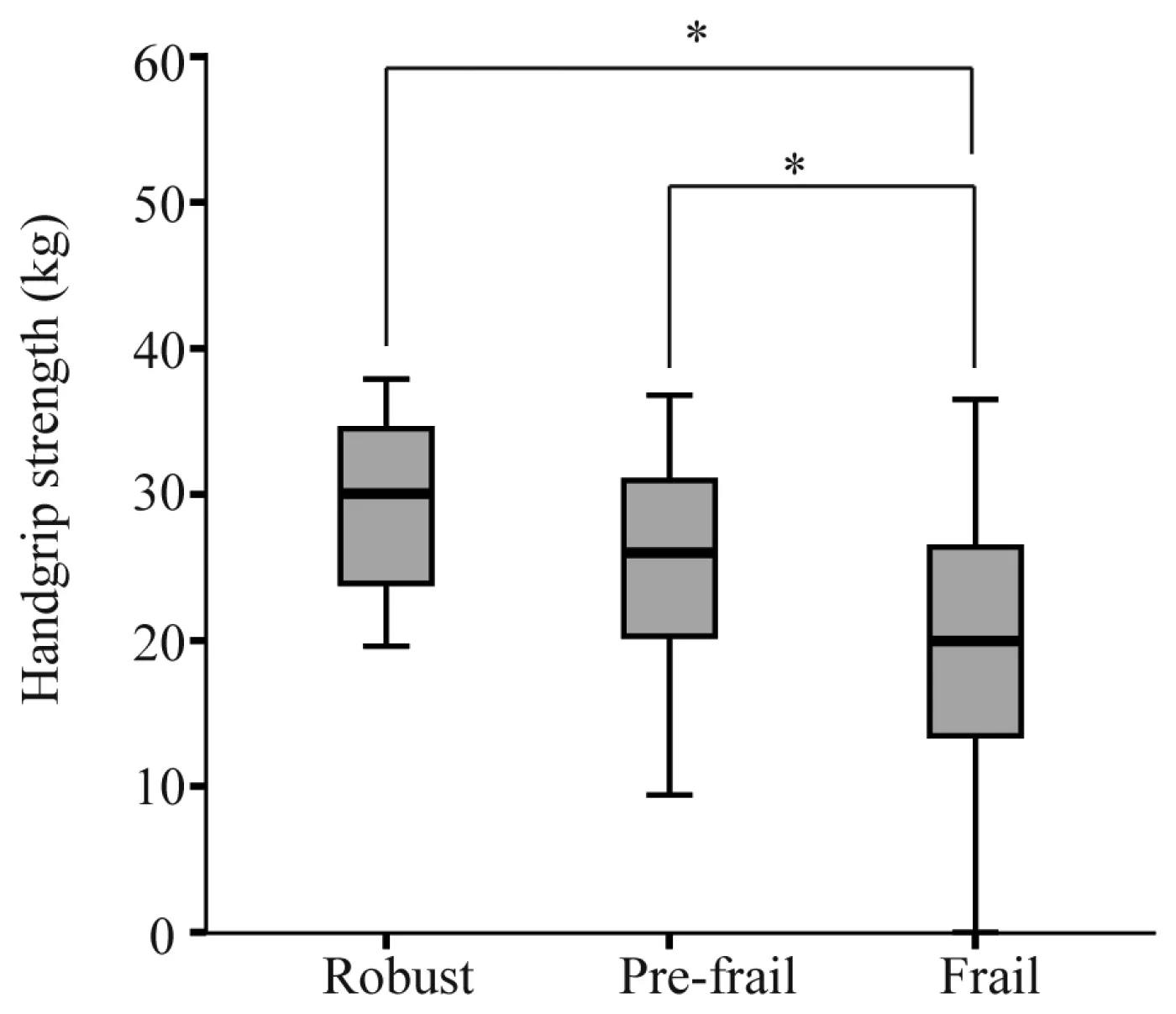
Comparison of handgrip strength between robust, prefrail, and frail patients. Frail patients with DCM had significantly lower handgrip strength than prefrail and robust patients (*P* < .05). *P < .05 for Bonferroni-adjusted pairwise comparisons.

Frail patients with DCM showed significantly lower handgrip strength than prefrail and robust patients (*P* < .05; Fig. 2). There were 22 robust patients (30.0 ± 7.5 kg), 32 prefrail patients (25.2 ± 7.1 kg), and 40 frail patients (19.5 ± 9.7 kg). There was no difference in handgrip strength between prefrail and robust patients.

### 3.2. Comorbidities

Handgrip strength did not differ significantly according to the presence or absence of anemia, diabetes, hypertension, hypercholesterolemia, altered HDL cholesterol, hypertriglyceridemia, dyslipidemia, low BMI, or a marital partner after Bonferroni correction (Table 3).

**Table 3:** Handgrip strength according to comorbidity status.

Comorbidities		n	Handgrip strength (kg) Median (interquartile range)	*P* value
Anemia	Yes	17	22.1 (14.5–26.9)	.122
	No	77	24.7 (19.6–31.8)	
Diabetes	Yes	31	20.8 (15.9–26.4)	.015
	No	63	25.9 (20.4–32.1)	
Hypertension	Yes	61	24.2 (17.5–30.9)	.312
	No	33	24.4 (20.7–32.3)	
Hypercholesterolemia	Yes	64	24.5 (18.8–30.9)	.846
	No	30	22.6 (18.2–32.1)	
Altered HDL cholesterol	Yes	41	26.7 (20.0–32.1)	.125
	No	53	22.7 (17.5–28.7)	
Hypertriglyceridemia	Yes	21	20.7 (14.5–25.9)	.085
	No	73	24.9 (19.9–31.4)	
Dyslipidemia	Yes	77	24.4 (17.5–31.0)	.791
	No	17	23.0 (21.3–27.9)	
Low weight BMI	Yes	31	21.9 (15.4–28.7)	.134
	No	63	24.9 (19.9–32.0)	
Marital status	With partner	85	24.0 (18.2–30.9)	.103
	No partner	9	30.0 (21.3–34.3)	

The Bonferroni-adjusted significance threshold was *P* < .0056 (.05/9).

BMI = body mass index, HDL = high density lipoprotein.

## 4. Discussion

This study demonstrates that handgrip strength is a useful and accessible indicator of overall physical condition in patients with DCM. Our findings revealed statistically significant correlations between handgrip strength and key clinical metrics, including gait speed, hand dexterity (as measured by the counter test and pinch strength), and total neurological function (JOA score). This is particularly evident when considering patients with and without walking difficulties. Our results are consistent with the strong association between handgrip strength and frailty, a concept well-documented in geriatric and other patient populations. Our study confirms that this relationship holds true for patients with DCM, where neurological deficits add a layer of complexity to the assessment of overall physical condition. It is crucial to interpret these findings within the context of a preoperative assessment. Handgrip strength in this cohort reflects the patients’ functional status before surgical intervention, making it a potentially useful component of presurgical risk stratification and for identifying patients who may benefit from prehabilitation.

A critical finding of this study was the potential clinical role of handgrip strength as a preoperative screening tool. Our work extends the previously reported link between handgrip strength and functional outcomes in other spinal conditions, such as lumbar spinal stenosis, where handgrip strength correlates with lower extremity function.^[12,13,22]^ Importantly, in the specific context of DCM, handgrip strength has been shown to be correlated with postoperative fall risk and functional outcomes in prospective studies, suggesting its value goes beyond simple correlation.^[16]^ The age- and sex-adjusted analyses provided a more nuanced interpretation of these associations. Handgrip strength remained significantly associated with LEP, counter test performance, and pinch strength after Bonferroni correction. In contrast, the association with walking speed was attenuated after adjustment and did not meet the corrected significance threshold. This finding suggests that age and sex may partly account for the crude association between handgrip strength and gait speed. In fact, the utility of combining handgrip strength with dexterity tests is already being explored for DCM diagnosis, supporting its role as a fundamental component of the assessment.^[14,15]^ This simple test could help clinicians quickly identify patients at an increased risk of poor functional status, which may necessitate prehabilitation strategies or adjustments to surgical planning. Notably, after applying the Bonferroni correction for multiple comparisons, the initial correlation with the nutritional marker GNRI and with comorbidities (e.g., diabetes) did not remain statistically significant, suggesting that these associations may be less robust than the primary correlation with motor function. While handgrip strength showed statistically significant correlations with gait speed (*r* = 0.617) and dexterity (*r* = 0.659), these correlations explain approximately 38% and 43% of the variance, respectively. This indicates that while handgrip strength is a valuable but nonspecific indicator, it does not capture the full complexity of motor dysfunction. Therefore, it should be considered a complementary tool to a comprehensive neurological and functional assessment, not a replacement for it.

The relationship between handgrip strength and frailty was a central theme in our results. Although the mFI-5 does not directly incorporate handgrip strength, our data showed a strong inverse relationship, with frail patients having a mean handgrip strength of 19.5 kg compared to 30.0 kg in robust patients. This reinforces the concept that handgrip strength is a fundamental component of frailty, regardless of the specific index used. While previous studies have shown that strength is associated with comorbidities such as diabetes,^[23]^ this association was not statistically significant in our cohort after correcting for multiple comparisons.

The present study has several limitations. First, its retrospective, single-center design may have introduced selection bias. Moreover, all participants were surgical candidates treated at a single institution. Therefore, the findings may not be generalizable to patients with milder DCM or those managed nonoperatively. The relatively small sample size, particularly the limited number of female participants, may also restrict the generalizability of the results. Second, because of the cross-sectional design, causal relationships could not be established, and longitudinal changes in handgrip strength, including changes after surgery, could not be evaluated. Third, although the partial correlation analyses adjusted for age and sex, residual confounding by symptom duration, baseline neurological severity, and radiographic characteristics, including the level and degree of cervical spinal cord compression, cannot be excluded. Future prospective studies should evaluate whether preoperative handgrip strength predicts postoperative recovery, quality of life, and falls in patients with DCM.

In conclusion, handgrip strength was associated with gait performance, hand dexterity, neurological function, and frailty in unadjusted analyses of surgical patients with DCM. After adjustment for age and sex, the strongest adjusted associations were observed for leg extension power and hand function. Handgrip strength may complement comprehensive preoperative neurological and functional assessment but should not be considered a substitute for it. Prospective studies are needed to determine its prognostic value for postoperative recovery, quality of life, and falls.

## Acknowledgments

The authors thank Mr. Nishikawa and the physical therapists at Shinkaminokawa Hospital for providing support in performing this study and Edanz (https://jp.edanz.com/ac) for editing the draft of this manuscript.

## Author contributions

**Conceptualization:** Hirokazu Inoue, Hideaki Sawamura, Yasuyuki Shiraishi, Atsushi Kimura, Katsushi Takeshita.

**Data curation:** Hirokazu Inoue, Hideaki Sawamura, Hitoshi Okami.

**Formal analysis:** Hirokazu Inoue, Hideaki Watanabe.

**Validation:** Hirokazu Inoue, Hideaki Watanabe.

**Visualization:** Hirokazu Inoue, Yasuyuki Shiraishi.

**Writing – original draft:** Hirokazu Inoue, Atsushi Kimura.

**Writing – review & editing:** Hirokazu Inoue.

**Supervision:** Katsushi Takeshita, Mitsuya Morita.
